# Familial history of heart disease and increased risk for elevated troponin in apparently healthy individuals

**DOI:** 10.1002/clc.23214

**Published:** 2019-06-07

**Authors:** Noa Cohen, Rafael Y. Brzezinski, Michal Ehrenwald, Itzhak Shapira, David Zeltser, Shlomo Berliner, Shani Shenhar‐Tsarfaty, Assi Milwidsky, Ori Rogowski

**Affiliations:** ^1^ Department of Internal Medicine “C”, “D” and “E”, Tel Aviv Sourasky Medical Center and Sackler Faculty of Medicine Tel Aviv University Tel Aviv Israel; ^2^ Department of Cardiology, Tel Aviv Sourasky Medical Center and Sackler Faculty of Medicine Tel Aviv University Tel Aviv Israel

**Keywords:** apparently healthy, family history, heart disease, high sensitive C reactive protein, high sensitive cardiac troponin

## Abstract

**Background:**

Family history of heart disease (FH‐HD) is associated with an increase drisk of subsequent HD. High sensitive cardiac troponin T (hs‐cTnT) is arecognized biomarker of myocyte injury even in HD free patients. We examined the association between FH‐HD and hs‐cTnT in apparently healthy individuals.

**Hypothesis:**

FH‐HD is associated with elevated hs‐cTnT in apparently healthy individuals.

**Methods:**

In a cross sectional study we analyzed data of apparently healthy individuals (n=3,821) recruited for the Tel‐Aviv Medical Center Inflammation Survey (TAMCIS). Blood samples were obtained for hs‐cTnT and high sensitive CRP (hs‐CRP) among other tests. FH‐HD was defined as first degree family member with HD diagnosis and classified as premature if the diagnosis was done before the age of 55 for men or 65 for women.

**Results:**

Elevated hs‐cTnT (>14 ng/L) was more common in FH‐HD of any age, and in premature FH‐HD (FH‐P‐HD) participants than in participants without FH‐HD (4.4% vs 2.0%, p<0.001 and 4.3% vs 2.0%, p=0.001, respectively). Adjustmentfor potential risk factors with association to elevated hs‐cTnT (age, sex, BMI, hypertension, diabetes, hs‐CRP, smoking and physical activity), showed that FH‐HD and FH‐P‐HD remained significantly associated with elevated hs‐cTnT (OR=1.62, p=0.025 and OR=1.70, p=0.039, respectively). Furthermore, we found that a significant interaction between FH‐HD or FH‐P‐HD and high levels ofhs‐CRP (>3 mg/L) increased the risk for elevated hs‐cTnT (OR=3.07, p=0.036 for FH‐HD and OR=3.25, p=0.053 for FH‐P‐HD).

**Conclusions:**

FH‐HD and its interaction with elevated hs‐CRP levels were significantly associated with elevated hs‐cTnT in apparently healthy individuals suggesting that an inflammatory process may be involved in this association.

## 1 INTRODUCTION

It has been shown that family history of cardiovascular disease (FH‐CVD) is associated with an increased risk of subsequent cardiovascular disease (CVD). In the Framingham offspring heart study which followed 2303 men and women free of CVD, the 8‐year incidence of CVD was higher in participants with at least one parent with premature CVD^1^ and increased even more in participants who had siblings with history of CVD.^2^ More recent studies showed greater severity of coronary artery disease at angiography in patients with family history of coronary heart disease (FH‐CHD)^3^ and demonstrated higher mortality risk with an excess in men with family history of myocardial infarction (MI).^4^ FH‐CHD was not included in the Framingham risk score for 10‐year coronary heart disease but has become part of other more recent risk algorithms.^5^, ^6^

With the aim of searching for the mechanism behind this association, different studies examined subclinical markers of CVD and their relationship with FH‐CHD in apparently healthy adults. Pandey et al conducted a systematic review and reported that independently of other risk factors, FH‐CHD, correlated with vascular function, inflammatory markers including C‐reactive protein (CRP), fibrinogen, and D‐dimer, and showed the strongest and most consistent association with coronary artery calcification and carotid intima thickness with 2‐3 approximate odds ratios.^7^

Sub‐clinical myocardial injury reflected by elevated high‐sensitivity cardiac troponin T and I (hs‐cTn T/I) is associated with increased cardiovascular risk in the general population,^8^, ^9^, ^10^ and in the obese, hypertensive, diabetic, and those with evident heart disease.^11^, ^12^, ^13^, ^14^, ^15^, ^16^, ^17^, ^18^ Moreover, serial measurements of changes in the hs‐cTnT levels over different periods of time were related to different incidences of coronary heart disease (CHD), heart failure (HF), and death years later.^8^, ^19^, ^20^ Interestingly, a study that investigated the mechanism underling these associations showed that high hs‐cTnT at baseline was associated with subclinical changes in terms of replacement fibrosis and increase in left ventricle (LV) mass 10 years later.^21^

The aim of this study was to examine the association between family history of heart disease (FH‐HD) and elevated hs‐cTnT as a sub‐clinical marker for heart disease (HD) in a population of apparently healthy individuals.

## 2 METHODS

### 2.1 Study population and study design

The Tel‐Aviv Medical Center Inflammation Survey (TAMCIS) is a registered databank of the Israeli Ministry of Justice as previously described.^22^, ^23^ The survey includes apparently healthy adults at the ages of 20 to 82 who have attended the Tel Aviv Medical Center as part of periodic tests. The assessment includes blood tests, physical examinations, a medical interview, and answering a detailed questionnaire.

In a cross sectional study design we analyzed the results of 3821 participants who were examined between September 2004 to July 2017. We excluded from the analysis subjects who had any CVD diagnosis—any coronary heart disease or stroke.

The study was approved by the local Ethics Committee (approval number 02‐049) and an informed consent was obtained from all participants.

### 2.2 Study variables

#### 2.2.1 Hs‐cTnT concentrations (dependent variable)

Blood samples were drawn from participants after 12 hours fast. Hs‐cTnT concentrations were measured using sandwich immunoassay technique by Roche Elecsys 2010 Analyzer (Roche Diagnostics). The assay detection capability ranges from 3 to 100 000 ng/L, values below the lower range border defined as 3 ng/L.^24^ Concentrations below 5 ng/L are measurable but less precise. Thus, the limit of detection is defined as 5 ng/L.^9^, ^19^ A concentration of 14 ng/L or higher represents the 97th percentile in the TAMCIS study sample and the 99th percentile value for a healthy reference population, and is referred to as elevated.^25^ Therefor hs‐cTnT was defined as a three‐level variable: hs‐cTnT ≤ 5 ng/L as undetectable, 5 ≤ hs‐cTnT<14 ng/L as detectable, and hs‐cTnT > 14 ng/L as elevated.

#### 2.2.2 Family history of HD (independent variable)

Participants filled‐in a questionnaire regarding their FH‐HD. They were asked to indicate whether they had a first degree family member who had HD and the age at diagnosis. Participants who answered these questions (n = 3821) were classified as having or not having FH‐HD. We defined history of premature HD as presence of a first degree family member who had a HD diagnosis before the age of 55 years for men and 65 for women and history of late HD (FH‐L‐HD) as presence of a family member who had a HD diagnosis later than that age.^1^, ^2^

#### 2.2.3 Other variables

Demographic information as sex and age, lifestyle habits as smoking and exercise, and use of regular medications were self‐reported by participants. Physical activity was defined as present or absent and classified by below or more than a mean of 2.5 hours per week. The participants were asked to indicate if they are currently cigarette smokers, ex‐smokers, or have never smoked. They were asked to indicate the number of cigarettes they smoke per day and at what age they started smoking. Participants currently smoking were defined as “smokers” and those who have never smoked or did smoke in the past were defined as “non‐smokers.” Rest pulse, blood pressure, height, waist, and weight were measured by the medical crew as part of the physical examination. Hypertension was defined as systolic blood pressure of 140 mm Hg or higher, diastolic blood pressure of 90 mm Hg or higher, or based on the use of antihypertensive medications. Body mass index (BMI) was calculated as the weight in kilograms divided by height in meters square. Diabetes mellitus was defined according to participants self‐report of physician diagnosis, or current use of diabetic medication, or hemoglobin A1C > 6.5%.

Metabolic syndrome was defined by the existence of at least three of the following factors: (a) Increased waist circumference (WC; men >102 cm, women >88 cm); (b) Triglycerides ≥1.65 mmol/L; (c) Low high density lipoprotein (HDL) cholesterol (men <1.03 mmol/L, women <1.29 mmol/L); (d) Hypertension (≥130/≥85 mm Hg); and (e) impaired fasting glucose (≥6.1 mmol/L 5.55mmo; l/l ATPIII).^26^

High sensitivity C‐reactive protein (hs‐CRP) levels were measured by using BN2 model nephelometer (Dade Behring, Cardio Phase hs‐CRP Assay, Marburg, Germany).^27^

We also defined hs‐CRP as categorical variable according to the cutoff point of 3 mg/L.

### 2.3 Statistical analysis

We performed descriptive statistics for variables as demographic data, blood test results, medical diagnoses, and life habits among all study population. *χ* ^2^ test was used to assess statistical significance of differences in hs‐cTnT levels between different categorical variables. *P* values of <.05 were considered statistically significant. We employed logistic regression with elevated troponin (hs‐cTnT > 14 ng/L) as the dependent variable, and examined the association of FH‐HD with hs‐cTnT when adjusting for other traditional risk factors for HD. Potential modifying effects on the association observed between FH‐HD or FH‐P‐HD with hs‐cTnT were analyzed. This was done by stratification, odds ratio calculations and heterogeneity testing using the Mantel‐Haenszel test. Relevant interactions were further included and tested in the multivariable analysis.

The IBM spss Statistics 22.0 statistical package and WinPepi version 11.65 were used to perform all statistical analyses (IBM Corporation, Armonk, New York).

## 3 RESULTS

Demographic and cardiac risk factor characteristics of the study population are presented in Table 1. The mean age of the 3821 participants was 48.6 (SD = 10.3), 2847 (74.5%) were males, 383 (10.0%) were smokers, and 1413 (37.0%) reported above 2.5 hours of physical activity per week. 637 (16.7%) of participants had a BMI greater than 30, 502 (13.1%) were previously diagnosed as hypertensive, 490 (12.8%) had metabolic syndrome, and 153 (4.0%) of participants were categorized as diabetics. Among 3821 participants, 1988 (52.0%) had undetectable levels of hs‐cTnT (≤5 ng/L), 1718 (45.0%) had detectable hs‐cTnT levels (5 < hs‐cTnT≤14 ng/L), and 115 (3%) had elevated levels (>14 ng/L). Elevated hs‐CRP (>3 mg/L) was found in 855 (22.4%) of participants. FH‐HD, FH‐P‐HD, and FH‐L‐HD were reported by 1635 (42.8%), 782 (20.5%), and 853 (22.3%) subjects, respectively. Information was missing on sex, age, physical activity, smoking, metabolic syndrome, BMI, hs‐CRP, and premature FH‐CHD for 2 (0.05%), 4 (0.1%), 291 (7.6%), 8 (2.0%), 15 (0.4%), 1 (0.03%), 5 (0.1%), and 107 (2.8%) participants, respectively.

**Table 1:** Characteristics of study sample (n = 3821^a^)

Characteristics	
Sex	
Male	2847 (74.5)
Female	972 (25.4)
Age, mean (SD)	48.6 (10.3)
Physical activity	
≤2.5 h/w	2117 (55.4)
>2.5 h/w	1413 (37.0)
Smoking	
No	3430 (89.8)
Yes	383 (10.0)
Metabolic syndrome	
No	3316 (86.8)
Yes	490 (12.8)
Hypertension	
No	3319 (86.9)
Yes	502 (13.1)
BMI	
≤30	3183 (83.3)
>30	637 (16.7)
Diabetes	
No	3668 (96.0)
Yes	153 (4.0)
hs‐CRP (mg/L)	
≤3	2961 (77.5)
>3	855 (22.4)
Family history of HD (any age)	
No	2186 (57.2)^b^
Yes	1635 (42.8)
History of premature HD	782 (20.5)
History of late HD	853 (22.3)
hs‐cTnT levels ( ng/L)	
Undetectable (≤5)	1988 (52.0)
Detectable (>5 and ≤14)	1718 (45.0)
Elevated (>14)	115 (3.0)

*Note*: BMI indicates body mass index, hs‐CRP indicates high sensitive c reactive protein, hs‐cTnT indicates high sensitive cardiac troponin T. Continues variables (age) are shown as mean (SD) and categorical variables are shown as number (%).

Abbreviations: FH‐HD, family history of heart disease; HD, heart disease.

a Information was missing on sex, age, physical activity, smoking, metabolic syndrome, BMI, Hs‐crp, and premature FH‐HD for 2 (0.05%), 4 (0.1%), 291 (7.6%), 8 (2.0%), 15 (0.4%), 1 (0.03%), 5 (0.1%), and 107 (2.8%) participants, respectively.

b Reference group for all groups of family history of HD.

Elevated hs‐cTnT was significantly more frequent among participants with FH‐HD, with FH‐P‐HD, or FH‐L‐HD compared to those without (4.4% vs 2.0%, *P* < .001), (4.3% vs 2.0%, *P* = .001), and (4.5% vs 2.0% *P* < .001), respectively. (Table 2). Male sex, older age, physical activity, metabolic syndrome, hypertension, and BMI above 30 and diabetes had a significant association with both detectable and elevated hs‐cTnT (Table 2).

**Table 2:** Univariate analysis of prevalence of hs‐cTnT levels by FH‐HD, premature FH‐HD, and other covariables

	Hs‐cTnT ≤ 5 ng/L		5 < Hs‐cTnT≤14 ng/L		Hs‐cTnT > 14 ng/L	
Characteristics	N = 1988	*P* value^a^	N = 1718	*P* value^a^	N = 115	*P* value^a^
Sex		<.001		<.001		<.001
Male	1266 (44.5%)		1477 (51.9%)		104 (3.7%)	
Female	721 (74.2%)		240 (24.7%)		11 (1.1%)	
Age—Mean (SD)						
	46.5 (9.9)		50.7 (10)		56.43 (10.5)	
Age band		<.001		<.001		<.001
Lower 33%	907 (63.3%)		501 (35.2%)		17 (1.2%)	
Mid 33%	651 (51.1%)		590 (46.3%)		33 (2.6%)	
Higher 33%	427 (38.2%)		626 (56.0%)		65 (5.8%)	
Physical activity		<.001		<.001		<.001
≤2.5 h/w	1206 (57.0%)		871 (41.1%)		40 (1.9%)	
>2.5 h/w	653 (46.2%)		696 (49.3%)		64 (4.5%)	
Smoking		.004		.007		.753
No	1758 (51.3%)		1569 (45.7%)		103 (3.0%)	
Yes	226 (59.0%)		147 (38.4%)		10 (2.6%)	
Metabolic syndrome		<.001		.004		<.001
No	1772 (53.4%)		1459 (44.0%)		85 (2.6%)	
Yes	210 (42.9%)		250 (51.0%)		30 (6.1%)	
Hypertension		<.001		<.001		<.001
No	1799 (54.2%)		1453 (43.8%)		67 (2.0%)	
Yes	189 (37.6%)		265 (52.8%)		48 (9.6%)	
BMI		<.001		.001		.008
≤30	1705 (53.6%)		1393 (43.8%)		85 (2.7%)	
>30	282 (44.3%)		325 (51.0%)		30 (4.7%)	
Diabetes		.001		.185		<.001
No	1929 (52.6%)		1641 (44.7%)		98 (2.7%)	
Yes	59 (38.6%)		77 (55.3%)		17 (11.1%)	
hs‐CRP ( mg/L)		.816		.391		.069
≤3	1537 (51.9%)		1343 (32.2%)		81 (2.7%)	
>3	448 (52.4%)		373 (43.6%)		34 (4.0%)	
FH‐HD (any age)		.028		.49		<.001
No	1171 (53.6%)		972 (44.5%)		43 (2.0%)	
Yes	817 (50.0%)		746 (45.6%)		72 (4.4%)	
FH‐P‐HD		.133		.706		.001
No	1171 (53.6%)		972 (44.5%)		43 (2.0%)	
Yes	394 (50.4%)		354 (45.3%)		34 (4.3%)	
FH‐L‐HD		.052		.465		<.001
No	1171 (53.6%)		972 (44.5%)		43 (2.0%)	
Yes	423 (49.6%)		392 (46.0%)		38 (4.5%)	

*Note*: BMI indicates body mass index, hs‐CRP indicates high sensitive c reactive protein. Continues variables are shown as mean (SD) and categorical variables are shown as number (%).

Abbreviations: hs‐cTnT, high sensitive cardiac troponin T; FH‐HD, family history of heart disease; FH‐L‐HD, late HD; FH‐P‐HD, premature FH‐HD; HD, heart disease.

a For comparison of hs‐cTnT level prevalence within subgroups of basic characteristics.

In a multivariable logistic regression model adjusting for age, sex, BMI, hs‐CRP, hypertension, diabetes, smoking, and physical activity, there was a significant association between FH‐HD (at any age) and elevated hs‐cTnT (odds ratio [OR] 1.62, 95% confidence interval [CI] [1.06‐2.46], *P* = .025) and between FH‐P‐HD and elevated hs‐cTnT (OR = 1.70, 95% CI [1.03‐2.82], *P* = .039) (Table 3).

**Table 3:** Multivariable models^a^ for the association between family history and premature family history of HD and hs‐cTnT > 14 ng/L

	Model 1		Model 2	
	OR (95% CI)	*P* value	OR (95% CI)	*P* value
Age	1.05 (1.03‐1.08)	<.001	1.05 (1.02‐1.08)	<.001
Sex (men)	3.11 (1.54‐6.28)	.002	2.59 (1.16‐5.79)	.021
Physical activity (>2.5 hours per week)	2.09 (1.37‐3.19)	.001	1.70 (1.03‐2.80)	.038
BMI (>30)	1.05 (0.64‐1.75)	.834	1.74 (0.98‐3.09)	.058
Smoking	1.20 (0.59‐2.45)	.62	0.86 (0.34‐2.22)	.766
hs‐CRP (>3 mg/L)	1.44 (0.90‐2.31)	.131	1.05 (0.59‐1.89)	.866
Diabetes	1.94 (1.01‐3.73)	.045	1.84 (0.82‐4.14)	.141
Hypertension	2.37 (1.48‐3.80)	<.001	2.37 (1.34‐4.21)	.003
FH‐HD any age	1.62 (1.06‐2.46)	.025	‐	‐
FH‐P‐HD	‐	‐	1.70 (1.03‐2.82)	.039

Abbreviations: BMI, body mass index; CI, confidence interval; FH‐HD, family history of heart disease; FH‐P‐HD, premature FH‐HD; HD, heart disease; hs‐CRP, high sensitive C‐reactive protein; hs‐cTnT, high sensitive cardiac troponin T; OR, odds ratio.

a Logistic regression for prediction of highly elevated hs‐cTnT (hs‐cTnT > 14 ng/L vs lower levels) by family history of CHD (model 1) and family history of premature CHD (model 2) while adjusting for other risk factors.

Table 4 summarizes the analysis of potential modifying effects in the association observed between FH‐HD or FH‐P‐HD with hs‐cTnT. Hs‐CRP was the only significant modifier of the associations between FH‐HD or FH‐P‐HD with hs‐cTnT (*P* for heterogeneity = .019 and .034, respectively). Stratification according to hs‐CRP levels revealed indeed much stronger ORs of FH‐HD (at any age) or of FH‐P‐HD with elevated hs‐cTnT, among participants with hs‐CRP levels of >3 than among participants with hs‐CRP levels of ≤3 (Table 4).

**Table 4:** Modifier analysis on the association between FH‐HD or FH‐P‐HD with hs‐cTnT

	FH‐HD (all ages)		FH‐P‐HD	
Characteristics	OR (95% CI)	*P* for heterogeneity	OR (95% CI)	*P* for heterogeneity
Sex		.407		.5
Male	2.48 (1.63‐3.83)		2.47 (1.47‐4.13)	
Female	1.46 (0.63‐2.32)		1.47 (0.23‐7.64)	
Age band		.669		.603
Lower 33%	1.59 (0.51‐4.65)		1.60 (0.36‐5.61)	
Mid 33%	2.43 (1.12‐5.60)		2.77 (1.10‐7.02)	
Higher 33%	1.66 (0.95‐2.95)		1.68 (0.83‐3.35)	
Physical activity		.273		.625
≤2.5 h/w	1.65 (0.84‐3.28)		1.85 (0.80‐4.09)	
>2.5 h/w	2.60 (1.50‐4.63)		2.36 (1.17‐4.68)	
Smoking		.458		.743
No	2.13 (1.40‐3.27)		2.21 (1.33‐3.66)	
Yes	3.59 (0.80‐21.76)		2.94 (0.38‐22.28)	
Metabolic syndrome		.25		.589
No	2.50 (1.22‐2.14)		2.37 (1.33‐4.18)	
Yes	1.50 (0.67‐3.49)		1.78 (0.64‐4.76)	
Hypertension		.464		.539
No	2.10 (1.25‐3.56)		2.11 (1.11‐3.95)	
Yes	1.55 (0.79‐3.17)		1.57 (0.69‐3.57)	
BMI		.222		.912
≤30	2.55 (1.60‐4.12)		2.17 (1.19‐3.91)	
>30	1.49 (0.66‐3.44)		2.05 (0.82‐5.13)	
Diabetes		.254		.308
No	2.40 (1.56‐3.75)		2.42 (1.43‐4.07)	
Yes	1.27 (0.41‐4.17)		1.18 (0.23‐5.05)	
hs‐CRP (mg/L)		.019		.034
≤3	1.72 (1.08‐2.76)		1.68 (0.92‐2.98)	
>3	5.22 (2.18‐14.33)		5.28 (1.91‐15.86)	

Abbreviations: BMI, body mass index; CI, confidence interval; FH‐HD, family history of heart disease; FH‐P‐HD, premature FH‐HD; hs‐CRP, high sensitive C‐reactive protein; hs‐cTnT, high sensitive cardiac troponin T; OR, odds ratio.

In view of these results, interaction variables of FH‐HD or of FH‐P‐HD with hs‐CRP levels were included in additional multivariable analysis models. (Table 5).

**Table 5:** Multivariable models^a^ for the association between family history and premature family history of HD and hs‐cTnT > 14 ng/L with supplementation of interaction variable

	Model 3		Model 4	
	OR (95% CI)	*P* value	OR (95% CI)	*P* value
Age	1.05 (1.03–1.08)	<.001	1.05 (1.02–1.08)	<.001
Sex (man)	3.08 (1.52‐6.23)	.002	2.49 (1.11‐5.57)	.027
Physical activity (>2.5 hours per week)	2.06 (1.35‐3.14)	.001	1.65 (1.00‐2.73)	.049
BMI (>30)	1.02 (0.61‐1.71)	.93	1.70 (0.95‐3.04)	.072
Smoking	1.12 (0.55‐2.31)	.754	0.82 (0.32‐2.10)	.672
hs‐CRP (>3 mg/L)	0.69 (0.28‐1.68)	.41	0.59 (0.24‐1.44)	.245
Diabetes	1.87 (0.97‐3.61)	.061	1.72 (0.76‐3.90)	.195
Hypertension	2.43 (1.51‐3.89)	<.001	2.44 (1.37‐4.33)	.002
FH‐HD any age	1.23 (0.76–1.99)	.4	‐	‐
FH‐HD any age by hs‐CRP	3.07 (1.08–8.77)	.036	‐	‐
FH‐P‐HD	‐	‐	1.25 (0.68–2.29)	.476
FH‐P‐HD by hs‐CRP	‐	‐	3.25 (0.99–10.69)	.053

Abbreviations: BMI, body mass index; CI, confidence interval; FH‐HD, family history of heart disease; FH‐P‐HD, premature FH‐HD; HD, heart disease; hs‐CRP, high sensitive C‐reactive protein; hs‐cTnT, high sensitive cardiac troponin T; OR, odds ratio.

a Logistic regression for prediction of highly elevated hs‐cTnT (hs‐cTnT > 14 ng/L vs lower levels) by family history of HD (model 3) and premature HD (model 4) while adjusting for other risk factors and interaction variable.

FH‐HD at any age was found to interact with hs‐CRP concentration in its association with elevated hs‐cTnT (OR 3.07, 95% CI [1.08‐8.77], *P* = .036 for the interaction variable) while the association of FH‐HD at any age alone with elevated hs‐cTnT became nonsignificant (OR 1.23, 95% CI [0.76‐1.99], *P* = .400) (Table 4). The interaction variable FH‐P‐HD with hs‐CRP yielded an adjusted OR of 3.25, 95% CI [0.99‐10.69], *P* = .053 rendering an OR of 1.25, 95% CI [0.68‐2.29], *P* = .476 for FH‐P‐HD alone (Table 5).

## 4 DISCUSSION

The main finding of the current study is the significant association between FH‐HD, premature and not premature, and elevated levels of hs‐cTnT. In the multivariable analysis this association was independent of several accepted risk factors of HD. Additionally, FH‐HD had a significant interaction with hs‐CRP in its association with elevated levels of hs‐cTnT. Elevated hs‐cTnT remained also significantly associated with male sex, age, hypertension, known risk factors for HD, and also linked with physical activity probably related to physiological changes in cell membrane of myocytes during exercise.^28^ A recent editorial and a review of 145 studies reported that both intensive long duration and also short or intermittent exercise not necessarily of high intensity can cause a rise in cardiac troponin. The authors concluded that the mechanism is most probably physiologic and a result of an enhanced release of cytosolic troponin follows increased membrane permeability. Production of reactive oxygen species, alternation in calcium, pH or glucose metabolism, and other cardiovascular stressors are suggested as potential factors involved in this physiologic reaction.^28^, ^29^

The mechanisms of the relationship between FH‐HD and subsequent HD events are not well known. As coronary heart disease is the most common heart disease, we focused our discussion on trying to understand the underlying factors of its association with family history of coronary heart disease (FH‐CHD). The relationship between FH‐CHD and subsequent CHD could be partially explained by both potential genetic and environmental factors. Familial hypercholesterolemia and familial combined hyperlipidemia are genetic disorders that increase the risk for CHD.^30^, ^31^ However, not only these specific and relatively rare syndromes have an important genetic component, but several biochemical processes associated with the pathogenesis of CHD can involve genetic disorders. Examples include lipid metabolism, inflammatory response, endothelial dysfunction, platelet function abnormalities, thrombosis, fibrinolysis, and blood pressure regulation.^32^ In a genome wide association study, loci related to early onset of CHD were found. The locus 9p21, that probably accounts for the encoding of controlling RNA, is the most common.^33^

Common exposure among family members to pollutants, similar microbiome, or shared lifestyle and cultural habits may also underlie the association between FH‐CHD and subsequent CHD events.

As hs‐cTnT is a marker of both clinical and subclinical CHD, the association between FH‐CHD and hs‐cTnT has biological rationality and may be utilized for better risk stratification of CHD in apparently healthy individuals.

To the best of our knowledge, studies on the association between FH‐CHD and hs‐cTnT were not published. Moreover, neither the association between hs‐cTnT and hs‐CRP or the interaction of FH‐CHD with hs‐CRP concentration in the prediction of high hs‐cTnT, were previously reported. This interaction may suggest involvement of an inflammation process indicated by increased hs‐cTnT in subjects with FH‐CHD and elevated levels of hs‐CRP. In a prospective study that examined the risk factors for CVD in participants to the Scottish Health Surveys who were followed over 7 years, the greatest risk of CVD was observed in participants with FH‐CVD and elevated CRP or hypertension.^34^

The current study has several limitations. This is a study in which the data were retrieved from an existing database leaving the risk for residual confounding. Information on FH‐HD or FH of premature HD was obtained from a questionnaire in which participants were asked to indicate, if relevant, the first degree family member and the age at diagnosis. The participants were not asked to indicate what the specific heart disease diagnosis is for them or for their family member. This information was not validated by medical documentation submitted by participants. In addition, we do not have long term clinical outcome data to assess the prognostic significance of the current findings. However, in light of past and recent large studies demonstrating the prognostic power of FH‐CHD and troponin elevation,^1^, ^35^, ^36^ it is probable that the combination of the two factors is patho‐physiologically related and has clinical significance.

We cannot entirely rule out the possibility of an effect of multiple comparisons within the analyses that we conducted. However, the various stratifications and the corresponding heterogeneity analyses were based on individual hypotheses regarding potential mechanisms responsible for the observed association between FH‐HD or FH‐P‐HD and hs‐cTnT allowing for these analyses without a necessary adjustment for multiple comparisons.^37^, ^38^

One strength of the study may rely on the fact that though having a cross‐sectional design, it is most probable that the independent variable (FH‐HD) preceded in time the dependent one (hs‐cTnT), and thus supporting causality for this association.

## 5 CONCLUSION

We found that FH‐HD and its interaction with hs‐CRP levels is associated with elevated hs‐cTnT, suggesting that an inflammatory process may be involved in this association. A combined assessment of FH‐HD, hs‐CRP, and hs‐cTnT could improve accuracy in prediction of future HD events. The findings deserve further confirmation in additional studies and future research regarding pathophysiology and clinical relevance.

## CONFLICT OF INTEREST

The authors declare no potential conflict of interests.
